# The Fulton County Medical Society

**Published:** 1871-04

**Authors:** 


### THE FULTON COUNTY MEDICAL SOCIETY.

The proceedings of this Society have been unavoidably left out of this issue. Our friends must bear with us.

